# Alcoholic cardiomyopathy–related mortality in the United States, 1999–2024: temporal trends and sociodemographic and geographic disparities

**DOI:** 10.3389/fcvm.2026.1972634

**Published:** 2026-10-12

**Authors:** Youming Jie, Weikai Chen, Dongmei Wu

**Affiliations:** 1 Department of Cardiology, Zhangzhou Affiliated Hospital of Fujian Medical University, Zhangzhou, Fujian, China; 2 Department of Emergency, Zhangzhou Affiliated Hospital of Fujian Medical University, Zhangzhou, Fujian, China; 3 Department of Anorectal Surgery, Zhangzhou People's Hospital, Zhangzhou, Fujian, China

**Keywords:** adult, alcoholic cardiomyopathy, CDC WONDER, mortality, trends

## Abstract

**Background:**

Alcoholic cardiomyopathy is associated with long-term heavy alcohol consumption and may progress to heart failure and death. This study evaluated temporal trends in alcoholic cardiomyopathy mortality among US adults aged ≥25 years from 1999 to 2024.

**Methods:**

Mortality data for US residents aged ≥25 years were obtained from the Centers for Disease Control and Prevention (CDC) WONDER database. Deaths with alcoholic cardiomyopathy (ICD-10 code I42.6) recorded as the underlying cause were identified. Death counts, age-adjusted mortality rates (AAMRs), crude death rates (CDRs), and 95% confidence intervals (CIs) were calculated. Joinpoint regression was used to estimate annual percentage changes (APCs) and average annual percentage changes (AAPCs), with analyses stratified by age group, sex, race/ethnicity, census region, and urbanization.

**Results:**

Overall, 13,710 deaths associated with alcoholic cardiomyopathy were recorded during 1999–2024. The AAMR declined from 0.31 (95% CI: 0.28–0.33) in 1999 to 0.22 (95% CI: 0.21–0.24) in 2024 (AAPC = −1.01%, 95% CI: −1.78 to −0.30). The decline was significant from 1999 to 2003 (APC = −8.80%, 95% CI: −19.36 to −2.88), followed by no significant change from 2003 to 2024 (APC = 0.54%, 95% CI: −0.01 to 1.43). Mortality was significantly higher in men than in women, and the male AAMR was significantly higher than the female AAMR. The male AAMR decreased from 0.63 to 0.32 (AAPC = −2.40%, 95% CI: −2.99 to −1.84), whereas the female AAMR changed from 0.07 to 0.08 (AAPC = 0.88%, 95% CI: −0.82 to 2.46). Among racial/ethnic groups, non-Hispanic Black individuals had the highest AAMR in 1999 (0.76), which decreased to 0.22 in 2024. The AAMR among non-Hispanic White individuals changed from 0.26 to 0.23 (AAPC = 0.98%, 95% CI: 0.17–1.84), while that among Hispanic individuals decreased from 0.33 to 0.10. The 55–64-year age group had the largest number of deaths and the highest CDR in 2024 (0.37). The West recorded the most deaths and the highest regional AAMR in 2024. In large metropolitan areas, the AAMR decreased from 0.34 in 1999 to 0.20 in 2020 (AAPC = −2.23%, 95% CI: −3.45 to −0.59).

**Conclusion:**

Alcoholic cardiomyopathy mortality decreased overall among US adults aged ≥25 years between 1999 and 2024, but significant sociodemographic and geographic differences remained. These findings support continued efforts to identify high-risk alcohol use, address alcohol use disorder, and detect cardiomyopathy early.

## 1 Introduction

Alcoholic cardiomyopathy is an acquired cardiomyopathy associated with long-term heavy alcohol consumption and may result in cardiac enlargement, impaired myocardial contractility, and progressive heart failure. As cardiac function may improve in some patients after reducing or discontinuing alcohol exposure, alcoholic cardiomyopathy is regarded as a potentially preventable and modifiable form of dilated cardiomyopathy (1). However, its diagnosis generally requires a history of prolonged alcohol exposure, evidence of structural and functional cardiac abnormalities, and exclusion of other causes of cardiomyopathy. Therefore, clinical recognition may be affected by underdiagnosis and variation in causal attribution (2).

Although alcoholic cardiomyopathy is not the most common alcohol-related cause of death, it represents a severe cardiovascular consequence of long-term alcohol exposure. Its mortality burden varies significantly across countries and regions, suggesting that drinking patterns, medical record systems, diagnostic practices, and the quality of death certification may contribute to differences in observed mortality (3). Previous studies from the United States (US) have shown that, despite its relative rarity, alcoholic cardiomyopathy has important clinical and public health implications, particularly when the disease progresses to advanced heart failure or death (4). A recent comprehensive review also described the fatal cardiovascular consequences associated with alcohol misuse, ranging from acute binge-related arrhythmias to chronic alcoholic cardiomyopathy and heart failure (5).

Estimation of the mortality burden associated with alcoholic cardiomyopathy is further complicated by differences in data sources and methods of case ascertainment. Manthey et al. reported discrepancies between civil registry data and modeled estimates of alcoholic cardiomyopathy mortality, particularly among older adults (6). In the US, long-term national mortality records provide an important source for evaluating population-level changes in mortality. However, systematic assessments of temporal, demographic, and geographic variation in alcoholic cardiomyopathy mortality in the US remain limited.

The CDC WONDER database provides standardized, population-based death certificate data for long-term national mortality surveillance in the US (7), while Joinpoint regression identifies changes in the slope of long-term mortality trends (8). Using these data, this study described deaths among US adults aged ≥25 years in which alcoholic cardiomyopathy was recorded as the underlying cause of death from 1999 to 2024 and examined mortality patterns according to sex, race/ethnicity, age group, US census region, and urbanization.

## 2 Materials and methods

### 2.1 Data source and study population

This descriptive population-level study used death certificate data from the CDC WONDER Underlying Cause of Death database to assess mortality among U.S. residents aged ≥25 years between 1999 and 2024. Deaths were included when alcoholic cardiomyopathy (ICD-10 code I42.6) was listed as the underlying cause of death. The analysis focused on temporal trends and subgroup differences at the population level and was not designed to establish individual-level causal relationships.

Because the study relied exclusively on de-identified, publicly available federal data, institutional review board approval was not required. The STROBE guidelines were followed to improve methodological transparency and rigor.

### 2.2 Data extraction

Mortality data were examined according to sex, race/ethnicity, age group, urbanization, and US census region (9). Race/ethnicity was classified as Hispanic or Latino, non-Hispanic (NH) White, or non-Hispanic (NH) Black or African American. Age groups were defined as 25–34, 35–44, 45–54, 55–64, 65–74, 75–84, and ≥85 years, with crude death rates (CDRs) used for age-specific analyses. Urbanization was classified according to the 2013 National Center for Health Statistics Urban–Rural Classification Scheme for Counties (10). Because of limitations in the comparability of the available urbanization classification, analyses by urbanization were restricted to 1999–2020. Geographic regions were defined according to US Census Bureau classifications as Northeast, Midwest, South, and West. Sex was classified as male or female.

### 2.3 Statistical analysis

Annual mortality trends were assessed using CDRs and age-adjusted mortality rates (AAMRs), expressed per 100,000 population with 95% confidence intervals (CIs). Rates were calculated by year and across sex, race/ethnicity, age group, state, census region, and urbanization status. CDRs were obtained by dividing the number of deaths for which alcoholic cardiomyopathy was the underlying cause by the corresponding population. AAMRs were derived by direct standardization to the 2000 US standard population. Temporal trends were evaluated using the National Cancer Institute Joinpoint Regression Program (version 5.1.0). Log-linear regression models were applied to estimate annual percentage changes (APCs) and average annual percentage changes (AAPCs), together with their corresponding 95% CIs (https://surveillance.cancer.gov/joinpoint/download). A trend was considered statistically significant when its slope differed significantly from zero based on a two-sided test. Statistical significance was defined as *P* < 0.05.

## 3 Results

### 3.1 Annual trends in overall mortality related to alcoholic cardiomyopathy

A total of 13,710 deaths among US adults aged ≥25 years were attributed to alcoholic cardiomyopathy as the underlying cause between 1999 and 2024. During this period, the overall AAMR declined from 0.31 (95% CI: 0.28–0.33) in 1999 to 0.22 (95% CI: 0.21–0.24) in 2024, corresponding to an AAPC of −1.01% (95% CI: −1.78 to −0.30). The Joinpoint analysis identified a significant decline between 1999 and 2003 (APC = −8.80%, 95% CI: −19.36 to −2.88), whereas the trend from 2003 to 2024 was not significant (APC = 0.54%, 95% CI: −0.01 to 1.43) (Table 1, Figure 1, Supplementary Table S1).

**Table 1:** Death counts and age-adjusted mortality rates or crude death rates per 100,000 population for alcoholic cardiomyopathy among US adults aged 25 years and older, stratified by age group, sex, race/ethnicity, census region, and urbanization.

Characteristic	Deaths	Sum of annual population	Rate in 1999 (95% CI)	Rate in 2024 (95% CI)	AAPC (95% CI)
**Overall**	13710	53,98,746349	0.31 (0.28 to 0.33)	0.22 (0.21 to 0.24)	−1.01 (−1.78 to −0.30)
Sex					
Male	11673	26,05,943716	0.63 (0.57 to 0.69)	0.32 (0.29 to 0.36)	−2.40 (−2.99 to −1.84)
Female	2037	27,92,802633	0.07 (0.05 to 0.09)	0.08 (0.07 to 0.10)	0.88 (−0.82 to 2.46)
Race/ethnicity					
Hispanic	985	74,25,20272	0.33 (0.23 to 0.46)	0.10 (0.07 to 0.15)	−3.49 (−4.71 to −2.20)
NH White	9781	36,73,762428	0.26 (0.23 to 0.29)	0.23 (0.21 to 0.26)	0.98 (0.17 to 1.84)
NH Black	2284	63,07,93625	0.76 (0.63 to 0.88)	0.22 (0.16 to 0.28)	−4.08 (−4.90 to −3.15)
Age group (CDR)					
25–34	467	11,03,082254	0.05 (0.03 to 0.08)	0.06 (0.04 to 0.08)	NA
35–44	1584	11,08,316424	0.21 (0.17 to 0.26)	0.15 (0.12 to 0.19)	−0.84 (−1.82 to −0.14)
45–54	3400	10,89,970438	0.41 (0.34 to 0.47)	0.18 (0.14 to 0.23)	−1.00 (−1.82 to −0.18)
55–64	4247	93,48,29484	0.59 (0.50 to 0.69)	0.37 (0.31 to 0.43)	−1.61 (−2.35 to −0.82)
65–74	2733	64,80,43148	0.56 (0.45 to 0.67)	0.34 (0.28 to 0.40)	−1.54 (−2.38 to 0.09)
75–84	1035	36,98,98963	0.41 (0.30 to 0.54)	0.30 (0.22 to 0.38)	−1.88 (−2.81 to −0.97)
85+	141	14,46,05638	NA(0.12 to 0.44)	0.17 (0.09 to 0.31)	NA
Census region					
Midwest	3001	11,58,892622	0.24 (0.20 to 0.29)	0.23 (0.19 to 0.28)	−0.49 (−1.69 to 0.77)
Northeast	2329	98,92,59418	0.28 (0.22 to 0.33)	0.16 (0.13 to 0.21)	−0.77 (−1.64 to 0.14)
South	4034	20,07,516980	0.31 (0.27 to 0.36)	0.17 (0.15 to 0.20)	−1.87 (−2.77 to −0.39)
West	4346	12,43,077329	0.46 (0.39 to 0.53)	0.26 (0.22 to 0.31)	−1.29 (−2.07 to 0.06)

Urbanization	Deaths	Population	AAMR in 1999 (95% CI)	AAMR in 2020 (95% CI)	AAPC(95% CI)
Large metropolitan	5483	24,72,137197	0.34 (0.30 to 0.37)	0.20 (0.18 to 0.23)	−2.23 (−3.45 to −0.59)
Medium/small metropolitan	3825	13,23,076625	0.30 (0.25 to 0.35)	0.34 (0.29 to 0.38)	0.59 (−0.49 to 1.81)
Rural counties	1993	67,86,34,169	0.30 (0.24 to 0.37)	0.34 (0.27 to 0.41)	0.02 (−1.43 to 1.58)

AAMR, age-adjusted mortality rate; AAPC, average annual percentage change; CDR, crude death rate; CI, confidence interval; NH, non-Hispanic; NA, not available or not reported.

**Figure 1:**
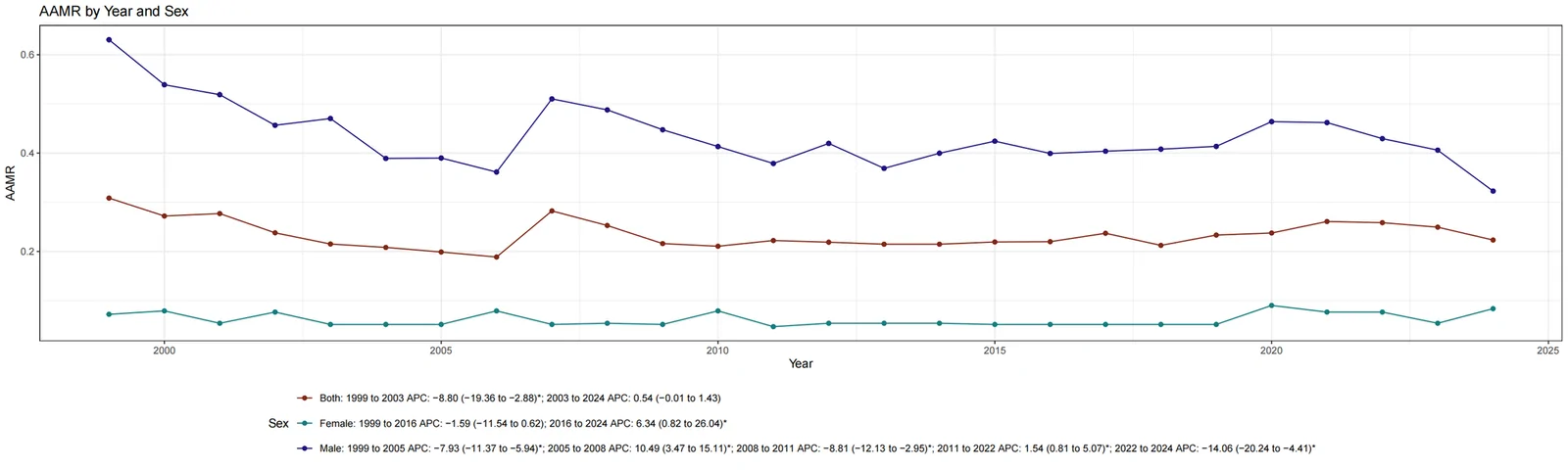
Temporal trends in overall and sex-stratified age-adjusted mortality rates for alcoholic cardiomyopathy among US adults aged ≥25 years, 1999–2024. AAMR, age-adjusted mortality rate; APC, annual percentage change; CI, confidence interval. *P < 0.05 denotes a statistically significant trend.

### 3.2 Trends stratified by sex

Men accounted for 11,673 deaths, compared with 2,037 among women, and had consistently higher AAMRs throughout the study period. The male AAMR showed an overall significant decline (AAPC = −2.40%, 95% CI: −2.99 to −1.84), with five distinct periods identified by Joinpoint regression. The rate decreased significantly during 1999–2005 (APC = −7.93%, 95% CI: −11.37 to −5.94), increased during 2005–2008 (APC = 10.49%, 95% CI: 3.47 to 15.11), and declined again during 2008–2011 (APC = −8.81%, 95% CI: −12.13 to −2.95). This was followed by a significant increase from 2011 to 2022 (APC = 1.54%, 95% CI: 0.81 to 5.07) and a significant decline from 2022 to 2024 (APC = −14.06%, 95% CI: −20.24 to −4.41). In women, the overall AAPC was 0.88% (95% CI: −0.82 to 2.46), indicating no significant long-term trend; however, the AAMR increased significantly during 2016–2024 (APC = 6.34%, 95% CI: 0.82 to 26.04) (Table 1, Figure 1, Supplementary Table S1).

### 3.3 Trends stratified by race

NH White individuals accounted for 9,781 deaths, followed by NH Black individuals with 2,284 and Hispanic individuals with 985. NH Black individuals had the highest AAMR in 1999, at 0.76 (95% CI: 0.63–0.88), which declined to 0.22 (95% CI: 0.16–0.28) by 2024. This group also had the largest overall decline, with an AAPC of −4.08% (95% CI: −4.90 to −3.15). Joinpoint analysis showed that the AAMR decreased sharply among NH Black individuals between 1999 and 2003 (APC = −15.27%, 95% CI: −28.63 to −7.88) and continued to decline at a slower rate from 2003 to 2024 (APC = −1.79%, 95% CI: −2.69 to −0.56).

Among Hispanic individuals, the AAMR decreased from 0.33 (95% CI: 0.23–0.46) in 1999 to 0.10 (95% CI: 0.07–0.15) in 2024, with an AAPC of −3.49% (95% CI: −4.71 to −2.20). In NH White individuals, the AAMR changed from 0.26 (95% CI: 0.23–0.29) to 0.23 (95% CI: 0.21–0.26), with an AAPC of 0.98% (95% CI: 0.17 to 1.84) (Table 1, Supplementary Table S2, Figure 2).

**Figure 2:**
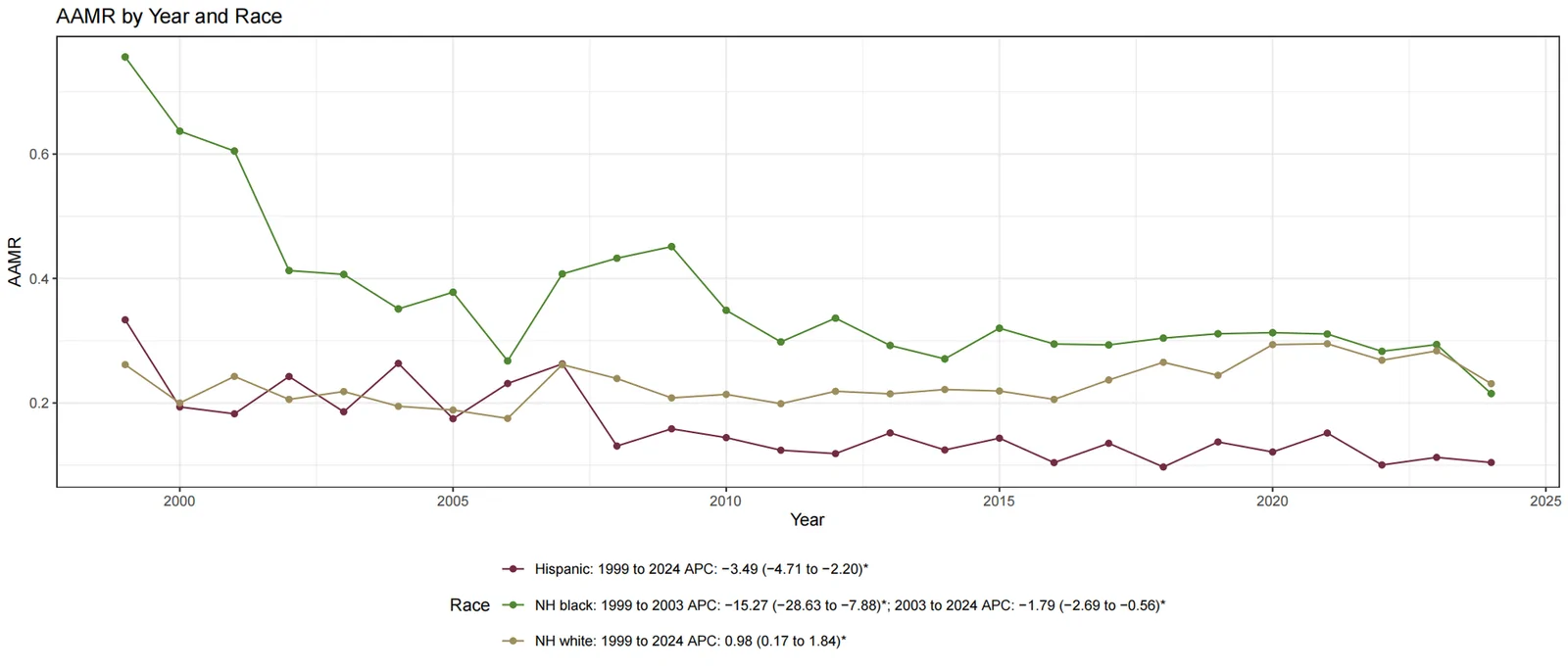
Temporal trends in age-adjusted mortality rates for alcoholic cardiomyopathy among US adults aged ≥25 years according to race/ethnicity, 1999–2024. AAMR, age-adjusted mortality rate; APC, annual percentage change; CI, confidence interval; NH, non-Hispanic. *P < 0.05 denotes a statistically significant trend.

### 3.4 Trends in crude death rates stratified by age group

Age-stratified analyses were based on CDRs. The 55–64-year age group accounted for the most deaths (4,247), followed by the 45–54-year group (3,400). The highest CDRs were observed in the 55–64-year group in both 1999 and 2024, declining from 0.59 (95% CI: 0.50–0.69) to 0.37 (95% CI: 0.31–0.43). The 65–74-year group ranked second in both years, with corresponding CDRs of 0.56 (95% CI: 0.45–0.67) and 0.34 (95% CI: 0.28–0.40).

Significant overall declines were observed in the 35–44-year group, from 0.21 to 0.15 (AAPC = −0.84%, 95% CI: −1.82 to −0.14), the 45–54-year group, from 0.41 to 0.18 (AAPC = −1.00%, 95% CI: −1.82 to −0.18), the 55–64-year group, from 0.59 to 0.37 (AAPC = −1.61%, 95% CI: −2.35 to −0.82), and the 75–84-year group, from 0.41 to 0.30 (AAPC = −1.88%, 95% CI: −2.81 to −0.97). Joinpoint regression analysis identified a marked increase in the 35–44-year group from 2014 to 2020 (APC = 14.98%, 95% CI: 9.98 to 31.39). Among those aged 55–64 years, the CDR increased gradually from 2004 to 2022 (APC = 1.15%, 95% CI: 0.61 to 3.45), followed by a significant decline through 2024 (APC = −12.89%, 95% CI: −20.25 to −1.80). Although all age groups, including 25–34 and ≥85 years, were included in the analysis, APC and AAPC estimates were not reported for these two groups because Joinpoint regression did not produce stable estimates (Table 1, Figure 3).

**Figure 3:**
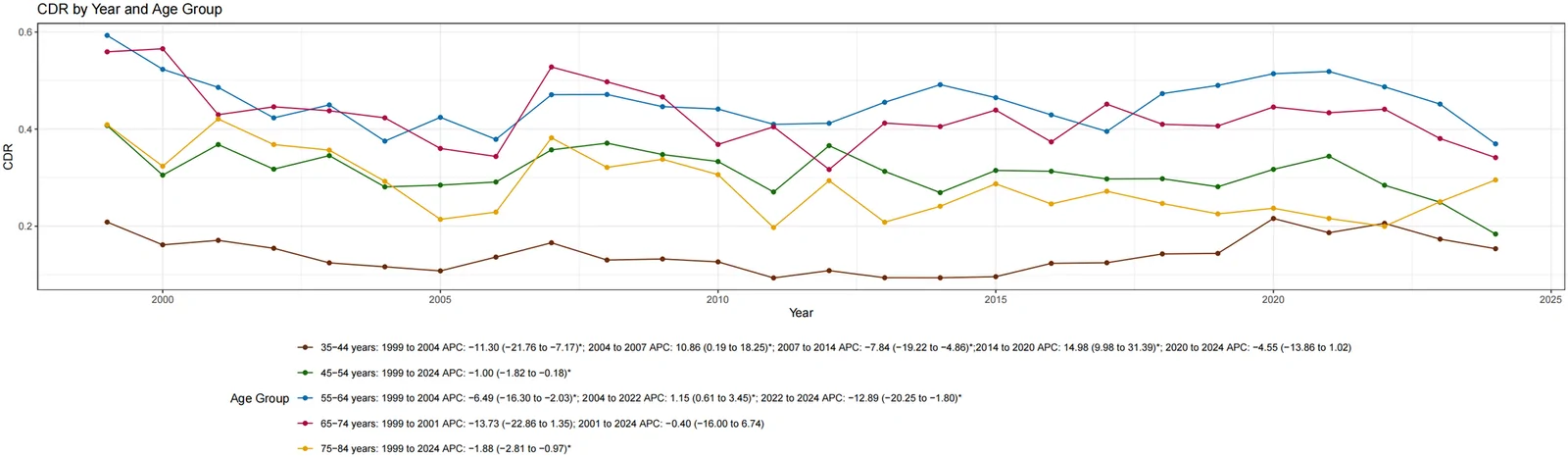
Age-specific temporal trends in crude death rates for alcoholic cardiomyopathy among US adults, 1999–2024. APC, annual percentage change; CDR, crude death rate; CI, confidence interval. *P < 0.05 denotes a statistically significant trend.

### 3.5 Trends stratified by geographic region

Across US census regions, the West recorded the highest number of deaths (4,346), followed by the South (4,034), Midwest (3,001), and Northeast (2,329). Regional differences were also evident in AAMR. In 1999, the West had the highest rate (0.46, 95% CI: 0.39–0.53), followed by the South (0.31, 95% CI: 0.27–0.36), Northeast (0.28, 95% CI: 0.22–0.33), and Midwest (0.24, 95% CI: 0.20–0.29). By 2024, the West remained highest at 0.26 (95% CI: 0.22–0.31), followed by the Midwest at 0.23 (95% CI: 0.19–0.28), the South at 0.17 (95% CI: 0.15–0.20), and the Northeast at 0.16 (95% CI: 0.13–0.21).

The South showed the largest overall decline (AAPC = −1.87%, 95% CI: −2.77 to −0.39), whereas the AAPCs were −1.29% (95% CI: −2.07 to 0.06) in the West, −0.77% (95% CI: −1.64 to 0.14) in the Northeast, and −0.49% (95% CI: −1.69 to 0.77) in the Midwest. Joinpoint analysis showed that in the South, the AAMR declined significantly between 1999 and 2003 (APC = −11.07%, 95% CI: −25.16 to −1.44) and remained stable thereafter. In the West, AAMR decreased significantly from 1999 to 2001 (APC = −17.20%, 95% CI: −25.24 to −0.67), then showed no significant change through 2024 (Table 1, Figure 4, Supplementary Table S3). State-level analysis similarly demonstrated geographic variation in AAMR (Figure 5, Supplementary Files).

**Figure 4:**
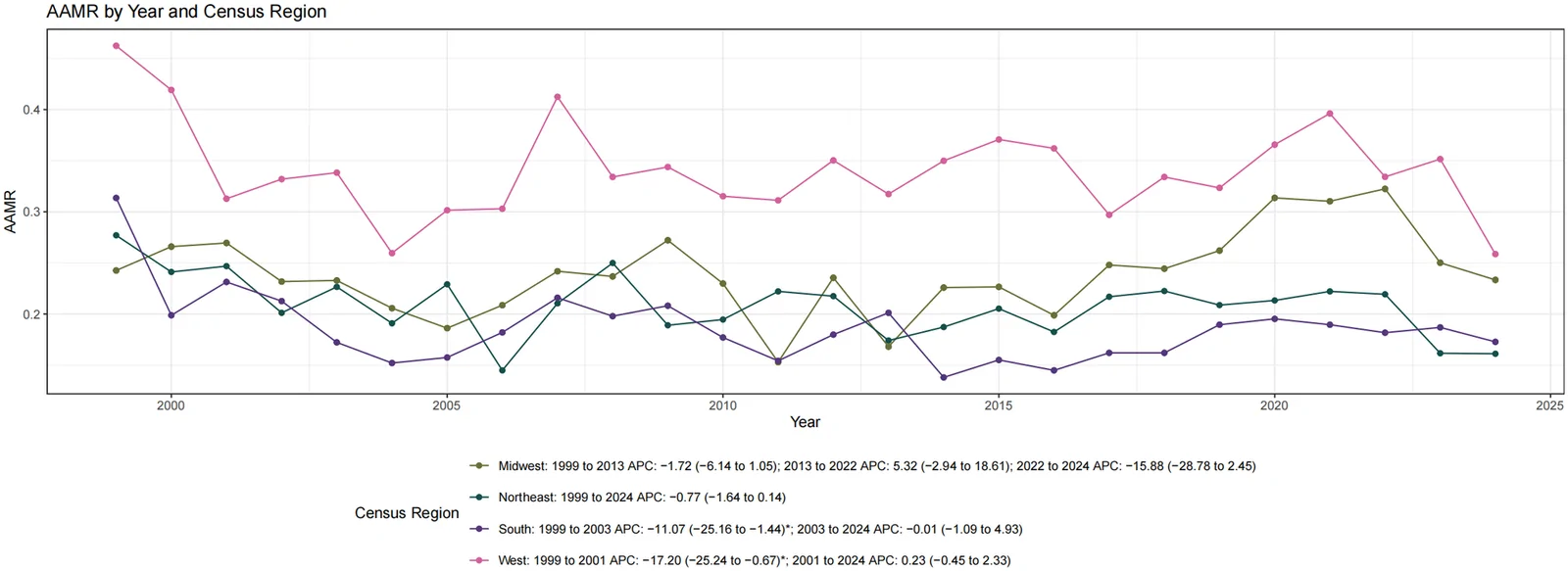
Temporal trends in age-adjusted mortality rates for alcoholic cardiomyopathy among US adults aged ≥25 years according to US census region, 1999–2024. AAMR, age-adjusted mortality rate; APC, annual percentage change; CI, confidence interval. *P < 0.05 denotes a statistically significant trend.

**Figure 5:**
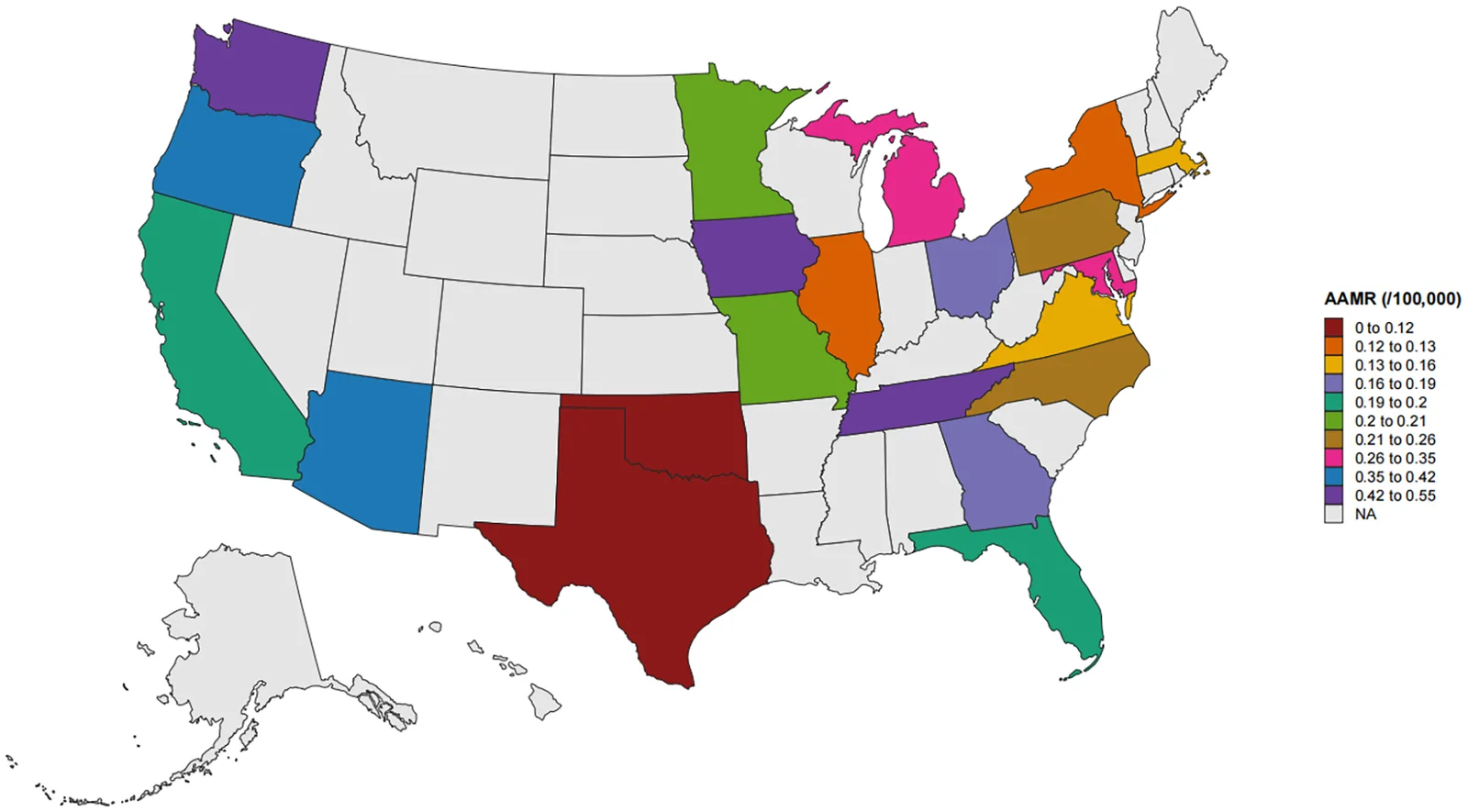
Geographic distribution of age-adjusted mortality rates for alcoholic cardiomyopathy across US states. AAMR, age-adjusted mortality rate; NA, not available.

Because of comparability limitations in the urbanization classification, urbanization-specific analyses were restricted to 1999–2020. Large metropolitan areas accounted for 5,483 deaths, compared with 3,825 in medium/small metropolitan areas and 1,993 in rural counties. The AAMR in large metropolitan areas declined from 0.34 (95% CI: 0.30–0.37) in 1999 to 0.20 (95% CI: 0.18–0.23) in 2020 (AAPC = −2.23%, 95% CI: −3.45 to −0.59). Rates increased from 0.30 (95% CI: 0.25–0.35) to 0.34 (95% CI: 0.29–0.38) in medium/small metropolitan areas (AAPC = 0.59%, 95% CI: −0.49 to 1.81) and from 0.30 (95% CI: 0.24–0.37) to 0.34 (95% CI: 0.27–0.41) in rural counties (AAPC = 0.02%, 95% CI: −1.43 to 1.58). Joinpoint regression identified a significant decline in large metropolitan areas between 1999 and 2004 (APC = −9.34%, 95% CI: −23.93 to −2.46), followed by a nonsignificant change through 2020. No significant temporal changes were observed in medium/small metropolitan areas or rural counties during 1999–2020 (Table 1, Figure 6, Supplementary Table S4).

**Figure 6:**
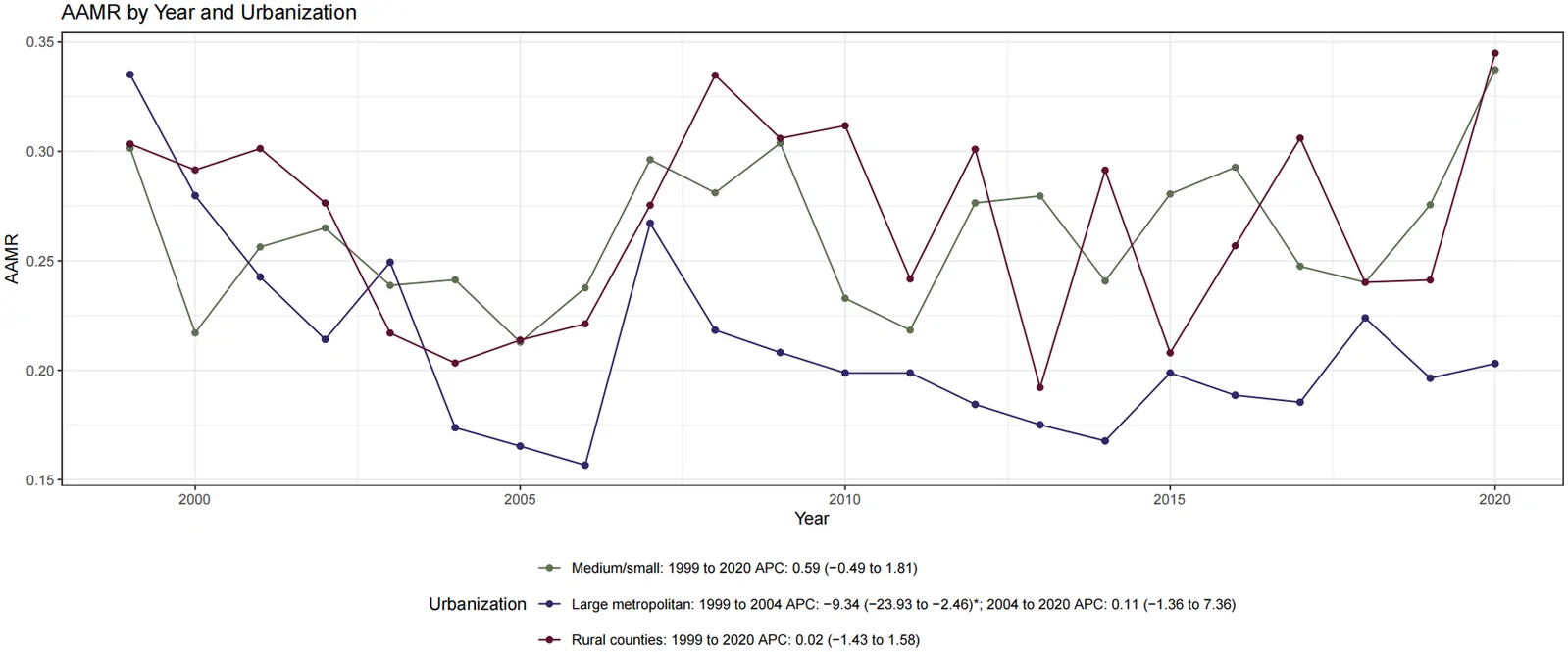
Temporal trends in age-adjusted mortality rates for alcoholic cardiomyopathy among US adults aged ≥25 years according to urbanization level, 1999–2020. AAMR, age-adjusted mortality rate; APC, annual percentage change; CI, confidence interval. *P < 0.05 denotes a statistically significant trend.

## 4 Discussion

Using national mortality data from CDC WONDER, this study characterized long-term temporal trends and sociodemographic and geographic differences in deaths among US adults aged ≥25 years for which alcoholic cardiomyopathy was recorded as the underlying cause between 1999 and 2024. Although alcoholic cardiomyopathy does not represent the most common alcohol-related cause of death, it remains a severe cardiovascular consequence of long-term alcohol exposure.

The observed mortality patterns should be considered in the broader context of alcohol-related mortality in the US. Rehm et al. suggested that the contribution of alcohol to cardiomyopathy mortality may be underestimated (11), while Manthey et al. demonstrated that recorded alcoholic cardiomyopathy mortality is significantly affected by drinking patterns and diagnostic recording (12). White et al. reported an overall increase in alcohol-related deaths in the US after 1999 (13), and changes in the prevalence of high-risk drinking and alcohol use disorder provide relevant exposure context for chronic alcohol-related conditions (14, 15). Alcohol-related mortality increased further during the COVID-19 pandemic (16), with Spillane et al. reporting significant increases in alcohol-induced mortality across age and race/ethnicity groups between 2000 and 2016 (17). These broader trends provide context for the fluctuations observed in this study but do not establish the factors responsible for changes in alcoholic cardiomyopathy mortality, particularly the early decline.

The pronounced decline between 1999 and 2003 requires careful interpretation. ICD-10 became the standard coding system for U.S. mortality beginning with deaths occurring in 1999; therefore, no ICD-9/ICD-10 transition occurred within the study period (18). Nevertheless, changes in coding, reporting, death-certification practices, or the attribution of alcohol as the underlying cause of death may have contributed to the early decline, in addition to a true epidemiologic decrease. Because these possibilities cannot be distinguished using the present descriptive mortality data, the decline from 1999 to 2003 should not be attributed to a single explanation.

Significant differences across population groups were also observed. Men accounted for the highest mortality burden throughout the study period, consistent with documented sex differences in alcohol exposure and alcohol-related mortality. At the same time, the significant increase in female mortality during the later study period indicates that temporal patterns were not uniform between sexes. Esser et al. reported sex differences in deaths due to excessive alcohol use, showing variation in both alcohol exposure and mortality risk (19). Differences across race/ethnicity may similarly arise from multiple factors, including drinking patterns, cardiovascular risk, access to medical care, and death certification practices (20). Changes in alcohol consumption during the COVID-19 pandemic may have further altered these patterns in some population groups (21).

Geographic and urban–rural differences further indicate that national mortality estimates alone may not capture priority areas for intervention. Mortality remained relatively high in the West, while changes were limited in medium/small metropolitan areas and rural counties. Regional differences in drinking environments, access to cardiovascular specialty care, and other contextual conditions may contribute to these patterns. Shahid et al. similarly reported associations between alcoholic cardiomyopathy mortality, social vulnerability, and geographic variation (22), while other evidence indicates that the mortality burden is unevenly distributed across populations and regions (23). Therefore, public health planning should consider both national trends and the needs of high-burden populations and areas.

The age-specific patterns also underscore the relevance of earlier intervention in the course of alcohol-related cardiac disease. Prolonged heavy alcohol consumption can cause cardiac chamber enlargement, impaired myocardial systolic function, and heart failure (24), with oxidative stress, mitochondrial injury, and neuroendocrine activation among the proposed mechanisms (25). Alcoholic cardiomyopathy commonly presents as dilated cardiomyopathy and is associated with poor prognosis (26). Alcohol abstinence remains the most important modifiable intervention (27), while the direct myocardial toxicity of ethanol further supports early recognition and follow-up (28). Previous studies have also reported alcohol-related injury to skeletal and cardiac muscle (29), and long-term benefit after alcohol withdrawal has been reported even among patients with severe disease (30). Therefore, early identification of alcohol use disorder, alcohol-focused intervention, and evidence-based heart failure management are relevant components of clinical care.

Several limitations should be considered when interpreting these findings. First, the descriptive population-level design allows identification of temporal trends and subgroup differences but does not establish causal relationships. Therefore, the present findings cannot determine whether alcohol exposure, clinical factors, healthcare-related factors, or policy changes caused the observed mortality trends. Second, case ascertainment was based on deaths for which ICD-10 code I42.6 was recorded as the underlying cause; deaths in which alcoholic cardiomyopathy appeared only as a contributing or secondary cause were not included, which may therefore lead to an underestimate of the broader burden of alcoholic cardiomyopathy-associated mortality. Third, death certificate data are vulnerable to underdiagnosis, misclassification, differences in certification practices, and incomplete documentation of alcohol exposure. Fourth, CDC WONDER lacks individual-level information on alcohol quantity and duration, cardiac function, comorbidities, treatment history, and other potential confounders, precluding adjusted causal analyses. Fifth, urbanization analyses were limited to 1999–2020, and estimates for some small subgroups may be unstable.

Despite these limitations, the data source also has important strengths. The CDC WONDER database provides nationwide coverage of death certificate data for U.S. residents over a 26-year period, with standardized ICD coding and age-adjusted mortality rates. The large number of mortality records allows comparisons across multiple demographic and geographic subgroups, while the public accessibility of the database facilitates reproducibility. Furthermore, Joinpoint regression enabled data-driven identification of changes in temporal slopes. The findings should therefore be interpreted as national surveillance estimates based on underlying-cause-coded mortality rather than comprehensive estimates of all deaths involving alcoholic cardiomyopathy or evidence of causal relationships.

## 5 Conclusion

Between 1999 and 2024, mortality with alcoholic cardiomyopathy recorded as the underlying cause declined overall among US adults aged ≥25 years, while significant differences remained across sex, age, race/ethnicity, region, and urbanization. These findings highlight the need for continued efforts to identify high-risk drinking, address alcohol use disorder, detect cardiomyopathy at an earlier stage, and provide evidence-based management to reduce preventable mortality.

## Data Availability

The data analyzed in this study are publicly available from the CDC WONDER database (https://wonder.cdc.gov/). Further inquiries can be directed to the corresponding author.
