# Supplementary material for: Alcoholic cardiomyopathy–related mortality in the United States, 1999–2024: temporal trends and sociodemographic and geographic disparities
Source: Front Cardiovasc Med. 2026 Oct 12;13:1972634. doi: 10.3389/fcvm.2026.1972634 (PMC13696187; doi:10.3389/fcvm.2026.1972634)
Supplement: Supplementary file 1 [file Datasheet2.docx]

**Supplementary Table S1.** Alcoholic cardiomyopathy-related age-adjusted mortality rates per 100,000 population, stratified by sex, among U.S. adults aged 25 years and older, 1999–2024.

| Year | **Age-Adjusted Mortality Rate (95% CI)** | | |
| --- | --- | --- | --- |
|  | **Overall** | **Male** | **Female** |
| 1999 | 0.31 (0.28-0.33) | 0.63 (0.57-0.69) | 0.07 (0.05-0.09) |
| 2000 | 0.27 (0.25-0.30) | 0.54 (0.49-0.59) | 0.08 (0.06-0.10) |
| 2001 | 0.28 (0.25-0.30) | 0.52 (0.47-0.57) | 0.05 (0.04-0.07) |
| 2002 | 0.24 (0.22-0.26) | 0.46 (0.41-0.50) | 0.08 (0.06-0.10) |
| 2003 | 0.22 (0.20-0.23) | 0.47 (0.42-0.52) | 0.05 (0.04-0.07) |
| 2004 | 0.21 (0.19-0.23) | 0.39 (0.35-0.43) | 0.05 (0.04-0.07) |
| 2005 | 0.20 (0.18-0.22) | 0.39 (0.35-0.43) | 0.05 (0.04-0.07) |
| 2006 | 0.19 (0.17-0.21) | 0.36 (0.32-0.40) | 0.08 (0.06-0.10) |
| 2007 | 0.28 (0.26-0.31) | 0.51 (0.46-0.56) | 0.05 (0.04-0.07) |
| 2008 | 0.25 (0.23-0.27) | 0.49 (0.44-0.53) | 0.05 (0.04-0.07) |
| 2009 | 0.22 (0.20-0.23) | 0.45 (0.41-0.49) | 0.05 (0.04-0.07) |
| 2010 | 0.21 (0.19-0.23) | 0.41 (0.37-0.45) | 0.08 (0.06-0.10) |
| 2011 | 0.22 (0.20-0.24) | 0.38 (0.34-0.42) | 0.05 (0.04-0.06) |
| 2012 | 0.22 (0.20-0.24) | 0.42 (0.38-0.46) | 0.05 (0.04-0.07) |
| 2013 | 0.21 (0.20-0.23) | 0.37 (0.33-0.41) | 0.05 (0.04-0.07) |
| 2014 | 0.21 (0.20-0.23) | 0.40 (0.36-0.44) | 0.05 (0.04-0.07) |
| 2015 | 0.22 (0.20-0.24) | 0.42 (0.38-0.46) | 0.05 (0.04-0.07) |
| 2016 | 0.22 (0.20-0.24) | 0.40 (0.36-0.44) | 0.05 (0.04-0.07) |
| 2017 | 0.24 (0.22-0.26) | 0.40 (0.37-0.44) | 0.05 (0.04-0.07) |
| 2018 | 0.21 (0.19-0.23) | 0.41 (0.37-0.45) | 0.05 (0.04-0.07) |
| 2019 | 0.23 (0.21-0.25) | 0.41 (0.38-0.45) | 0.05 (0.04-0.07) |
| 2020 | 0.24 (0.22-0.26) | 0.46 (0.42-0.50) | 0.09 (0.07-0.11) |
| 2021 | 0.26 (0.24-0.28) | 0.46 (0.42-0.50) | 0.08 (0.06-0.10) |
| 2022 | 0.26 (0.24-0.28) | 0.43 (0.39-0.47) | 0.08 (0.06-0.10) |
| 2023 | 0.25 (0.23-0.27) | 0.41 (0.37-0.44) | 0.05 (0.04-0.07) |
| 2024 | 0.22 (0.21-0.24) | 0.32 (0.29-0.36) | 0.08 (0.07-0.10) |

Abbreviations: CI, confidence interval.

**Supplementary Table S2.** Alcoholic cardiomyopathy-related age-adjusted mortality rates per 100,000 population, stratified by race/ethnicity, among U.S. adults aged 25 years and older, 1999–2024.

| Year | **Age-Adjusted Mortality Rate (95% CI)** | | |
| --- | --- | --- | --- |
|  | **NH Black or African American** | **NH White** | **Hispanic or Latino** |
| 1999 | 0.76 (0.63-0.88) | 0.26 (0.23-0.29) | 0.33 (0.23-0.46) |
| 2000 | 0.64 (0.52-0.75) | 0.20 (0.18-0.22) | 0.19 (0.12-0.29) |
| 2001 | 0.60 (0.49-0.72) | 0.24 (0.22-0.27) | 0.18 (0.12-0.27) |
| 2002 | 0.41 (0.33-0.52) | 0.21 (0.18-0.23) | 0.24 (0.17-0.34) |
| 2003 | 0.41 (0.32-0.50) | 0.22 (0.19-0.24) | 0.19 (0.12-0.27) |
| 2004 | 0.35 (0.27-0.45) | 0.19 (0.17-0.22) | 0.26 (0.18-0.37) |
| 2005 | 0.38 (0.30-0.47) | 0.19 (0.17-0.21) | 0.17 (0.12-0.25) |
| 2006 | 0.27 (0.20-0.34) | 0.18 (0.15-0.20) | 0.23 (0.16-0.32) |
| 2007 | 0.41 (0.33-0.50) | 0.26 (0.23-0.29) | 0.26 (0.19-0.36) |
| 2008 | 0.43 (0.35-0.52) | 0.24 (0.21-0.26) | 0.13 (0.09-0.19) |
| 2009 | 0.45 (0.36-0.54) | 0.21 (0.19-0.23) | 0.16 (0.11-0.22) |
| 2010 | 0.35 (0.27-0.44) | 0.21 (0.19-0.24) | 0.14 (0.09-0.21) |
| 2011 | 0.30 (0.23-0.38) | 0.20 (0.18-0.22) | 0.12 (0.08-0.18) |
| 2012 | 0.34 (0.27-0.42) | 0.22 (0.20-0.24) | 0.12 (0.08-0.18) |
| 2013 | 0.29 (0.23-0.37) | 0.21 (0.19-0.24) | 0.15 (0.11-0.21) |
| 2014 | 0.27 (0.21-0.34) | 0.22 (0.20-0.24) | 0.12 (0.09-0.18) |
| 2015 | 0.32 (0.25-0.40) | 0.22 (0.20-0.24) | 0.14 (0.10-0.20) |
| 2016 | 0.29 (0.23-0.37) | 0.21 (0.18-0.23) | 0.10 (0.07-0.15) |
| 2017 | 0.29 (0.23-0.37) | 0.24 (0.21-0.26) | 0.14 (0.09-0.19) |
| 2018 | 0.30 (0.24-0.38) | 0.27 (0.24-0.29) | 0.10 (0.07-0.14) |
| 2019 | 0.31 (0.25-0.39) | 0.24 (0.22-0.27) | 0.14 (0.10-0.18) |
| 2020 | 0.31 (0.25-0.39) | 0.29 (0.26-0.32) | 0.12 (0.09-0.16) |
| 2021 | 0.31 (0.25-0.39) | 0.30 (0.27-0.32) | 0.15 (0.11-0.20) |
| 2022 | 0.28 (0.22-0.35) | 0.27 (0.24-0.30) | 0.10 (0.07-0.14) |
| 2023 | 0.29 (0.23-0.37) | 0.28 (0.26-0.31) | 0.11 (0.08-0.16) |
| 2024 | 0.22 (0.16-0.28) | 0.23 (0.21-0.26) | 0.10 (0.07-0.15) |

Abbreviations: CI, confidence interval; NH, non-Hispanic.

**Supplementary Table S3.** Alcoholic cardiomyopathy-related age-adjusted mortality rates per 100,000 population, stratified by U.S. census region, among U.S. adults aged 25 years and older, 1999–2024.

| Year | **Age-Adjusted Mortality Rate (95% CI)** | | | |
| --- | --- | --- | --- | --- |
|  | **Northeast** | **Midwest** | **South** | **West** |
| 1999 | 0.28 (0.22-0.33) | 0.24 (0.20-0.29) | 0.31 (0.27-0.36) | 0.46 (0.39-0.53) |
| 2000 | 0.24 (0.19-0.30) | 0.27 (0.22-0.32) | 0.20 (0.16-0.23) | 0.42 (0.35-0.49) |
| 2001 | 0.25 (0.20-0.30) | 0.27 (0.22-0.32) | 0.23 (0.19-0.27) | 0.31 (0.26-0.37) |
| 2002 | 0.20 (0.16-0.25) | 0.23 (0.19-0.28) | 0.21 (0.18-0.25) | 0.33 (0.28-0.39) |
| 2003 | 0.23 (0.18-0.28) | 0.23 (0.19-0.28) | 0.17 (0.14-0.20) | 0.34 (0.28-0.39) |
| 2004 | 0.19 (0.15-0.24) | 0.21 (0.17-0.25) | 0.15 (0.12-0.18) | 0.26 (0.21-0.31) |
| 2005 | 0.23 (0.18-0.28) | 0.19 (0.15-0.23) | 0.16 (0.13-0.19) | 0.30 (0.25-0.35) |
| 2006 | 0.14 (0.11-0.19) | 0.21 (0.17-0.26) | 0.18 (0.15-0.21) | 0.30 (0.25-0.35) |
| 2007 | 0.21 (0.17-0.26) | 0.24 (0.20-0.29) | 0.22 (0.18-0.25) | 0.41 (0.35-0.47) |
| 2008 | 0.25 (0.20-0.30) | 0.24 (0.19-0.28) | 0.20 (0.17-0.23) | 0.33 (0.28-0.39) |
| 2009 | 0.19 (0.15-0.24) | 0.27 (0.22-0.32) | 0.21 (0.18-0.24) | 0.34 (0.29-0.40) |
| 2010 | 0.19 (0.15-0.24) | 0.23 (0.19-0.27) | 0.18 (0.15-0.21) | 0.32 (0.26-0.37) |
| 2011 | 0.22 (0.17-0.28) | 0.15 (0.12-0.19) | 0.15 (0.13-0.18) | 0.31 (0.26-0.36) |
| 2012 | 0.22 (0.17-0.27) | 0.24 (0.19-0.28) | 0.18 (0.15-0.21) | 0.35 (0.30-0.40) |
| 2013 | 0.17 (0.14-0.22) | 0.17 (0.13-0.21) | 0.20 (0.17-0.23) | 0.32 (0.27-0.37) |
| 2014 | 0.19 (0.15-0.24) | 0.23 (0.18-0.27) | 0.14 (0.11-0.16) | 0.35 (0.30-0.40) |
| 2015 | 0.21 (0.16-0.25) | 0.23 (0.18-0.27) | 0.16 (0.13-0.18) | 0.37 (0.32-0.42) |
| 2016 | 0.18 (0.14-0.23) | 0.20 (0.16-0.24) | 0.14 (0.12-0.17) | 0.36 (0.31-0.41) |
| 2017 | 0.22 (0.17-0.27) | 0.25 (0.20-0.29) | 0.16 (0.14-0.19) | 0.30 (0.25-0.34) |
| 2018 | 0.22 (0.18-0.27) | 0.24 (0.20-0.29) | 0.16 (0.14-0.19) | 0.33 (0.28-0.39) |
| 2019 | 0.21 (0.17-0.26) | 0.26 (0.21-0.31) | 0.19 (0.16-0.22) | 0.32 (0.27-0.37) |
| 2020 | 0.21 (0.17-0.26) | 0.31 (0.26-0.36) | 0.20 (0.17-0.23) | 0.37 (0.31-0.42) |
| 2021 | 0.22 (0.18-0.27) | 0.31 (0.26-0.37) | 0.19 (0.16-0.22) | 0.40 (0.35-0.45) |
| 2022 | 0.22 (0.18-0.27) | 0.32 (0.27-0.38) | 0.18 (0.15-0.21) | 0.33 (0.29-0.39) |
| 2023 | 0.16 (0.13-0.20) | 0.25 (0.21-0.30) | 0.19 (0.16-0.22) | 0.35 (0.30-0.40) |
| 2024 | 0.16 (0.13-0.21) | 0.23 (0.19-0.28) | 0.17 (0.15-0.20) | 0.26 (0.22-0.31) |

Abbreviations: CI, confidence interval.

**Supplementary Table S4.** Alcoholic cardiomyopathy-related age-adjusted mortality rates per 100,000 population, stratified by urbanization, among U.S. adults aged 25 years and older, 1999–2020.

| Year | **Age-Adjusted Mortality Rate (95% CI)** | | |
| --- | --- | --- | --- |
|  | **Large Metropolitan** | **Medium/Small Metropolitan** | **Rural/Counties** |
| 1999 | 0.34 (0.30-0.37) | 0.30 (0.25-0.35) | 0.30 (0.24-0.37) |
| 2000 | 0.28 (0.25-0.31) | 0.22 (0.18-0.26) | 0.29 (0.24-0.36) |
| 2001 | 0.24 (0.21-0.27) | 0.26 (0.22-0.30) | 0.30 (0.24-0.37) |
| 2002 | 0.21 (0.19-0.24) | 0.27 (0.22-0.31) | 0.28 (0.22-0.35) |
| 2003 | 0.25 (0.22-0.28) | 0.24 (0.20-0.28) | 0.22 (0.17-0.28) |
| 2004 | 0.17 (0.15-0.20) | 0.24 (0.20-0.28) | 0.20 (0.16-0.26) |
| 2005 | 0.17 (0.14-0.19) | 0.21 (0.18-0.25) | 0.21 (0.17-0.27) |
| 2006 | 0.16 (0.13-0.18) | 0.24 (0.20-0.28) | 0.22 (0.17-0.28) |
| 2007 | 0.27 (0.24-0.30) | 0.30 (0.25-0.34) | 0.28 (0.22-0.34) |
| 2008 | 0.22 (0.19-0.25) | 0.28 (0.24-0.32) | 0.33 (0.27-0.40) |
| 2009 | 0.21 (0.18-0.23) | 0.30 (0.26-0.35) | 0.31 (0.24-0.38) |
| 2010 | 0.20 (0.17-0.23) | 0.23 (0.20-0.27) | 0.31 (0.25-0.37) |
| 2011 | 0.20 (0.17-0.22) | 0.22 (0.18-0.26) | 0.24 (0.19-0.30) |
| 2012 | 0.18 (0.16-0.21) | 0.28 (0.24-0.32) | 0.30 (0.24-0.37) |
| 2013 | 0.18 (0.15-0.20) | 0.28 (0.24-0.32) | 0.19 (0.15-0.24) |
| 2014 | 0.17 (0.15-0.19) | 0.24 (0.20-0.28) | 0.29 (0.23-0.36) |
| 2015 | 0.20 (0.17-0.22) | 0.28 (0.24-0.32) | 0.21 (0.17-0.26) |
| 2016 | 0.19 (0.16-0.21) | 0.29 (0.25-0.34) | 0.26 (0.21-0.32) |
| 2017 | 0.19 (0.16-0.21) | 0.25 (0.21-0.29) | 0.31 (0.24-0.37) |
| 2018 | 0.22 (0.20-0.25) | 0.24 (0.20-0.28) | 0.24 (0.19-0.30) |
| 2019 | 0.20 (0.17-0.22) | 0.28 (0.24-0.31) | 0.24 (0.19-0.30) |
| 2020 | 0.20 (0.18-0.23) | 0.34 (0.29-0.38) | 0.34 (0.27-0.41) |

Abbreviations: CI, confidence interval.
